# Alterations of Epigenetic Regulators in Pancreatic Cancer and Their Clinical Implications

**DOI:** 10.3390/ijms17122138

**Published:** 2016-12-19

**Authors:** Brittany R. Silverman, Jiaqi Shi

**Affiliations:** Department of Pathology, School of Medicine, University of Michigan, Ann Arbor, MI 48109, USA; bsilv@umich.edu

**Keywords:** genetic alterations, epigenetic regulators, pancreatic neoplasms, pancreatic cancer, clinical implication

## Abstract

Pancreatic cancer is one of the most aggressive human cancer types with a five-year survival less than 7%. Emerging evidence revealed that many genetic alterations in pancreatic cancer target epigenetic regulators. Some of these mutations are driver mutations in cancer development. Several most important mechanisms of epigenetic regulations include DNA methylation, histone modifications (methylation, acetylation, and ubiquitination), chromatin remodeling, and non-coding ribonucleic acids (RNAs). These modifications can alter chromatin structure and promoter accessibility, and thus lead to aberrant gene expression. However, exactly how these alterations affect epigenetic reprogramming in pancreatic cancer cells and in different stages of tumor development is still not clear. This mini-review summarizes the current knowledge of epigenetic alterations in pancreatic cancer development and progression, and discusses the clinical applications of epigenetic regulators as diagnostic biomarkers and therapeutic targets in pancreatic cancer.

## 1. Introduction

Pancreatic cancer is the third leading cause of cancer death and is predicted to be the second leading cause in a decade in the United States. Despite more than 50 years of research and therapeutic development, pancreatic cancer remains to have a median survival rate of six months and a five-year overall survival around 6%–7%. Therefore, there is an urgent need to better understand the mechanisms of pancreatic cancer development so that new biomarkers for early diagnosis, prognosis, and therapeutic targets can be discovered.

Several whole genomic sequencing and copy number analysis studies of pancreatic ductal adenocarcinoma (PDA) recently discovered many altered signaling pathways [1,2,3]. Most of the studies have been focused on genetic abnormalities of driver mutations that occur in more than 50% of the PDAs, such as *KRAS*, *TP53*, *SMAD4*, and *CDKN2A*. The other lower prevalence mutated genes include those involved in Deoxyribonucleic acid [4] damage repair, chromatin remodeling, Wingless-type MMTV integration site family (WNT) signaling, transforming growth factor β signaling, Hedgehog signaling, and cell cycle regulation pathways.

Evidence emerged recently that many genetic alterations in pancreatic cancer target epigenetic regulators [5]. Epigenetic modifications are heritable changes in gene expression that do not alter DNA sequence. These modifications are referred to as non-DNA sequence-based heritability because they are remembered with cell divisions. Some of the most important mechanisms of epigenetic regulations include DNA methylation, histone modification (methylation, acetylation, phosphorylation, ubiquitination, and sumoylation), chromatin remodeling, and non-coding ribonucleic acids (RNAs). These modifications can alter chromatin structure and promoter accessibility, and thus lead to aberrant gene expression. Studies have shown that epigenetic alterations in cancer cells contribute to tumor initiation, progression, and metastasis. Whole genomic sequencing studies have also revealed driver mutations in epigenetic regulators in human cancers. While some of the mutations are oncogenic (i.e., *IDH1/2*, *EZH2*, and *DNMT3A*), others are tumor suppressive (i.e., *KDM6A*, *CREBBP/EP300*, and *SMARCB1*) [6]. In pancreatic cancer, recent whole genomic sequencing also revealed pathogenic mutations and structural variants in several epigenetic regulator genes including *KDM6A*, *ARID1A*, *ARID1B*, *PBRM1*, *SMARCA2*, *SMARCA4*, and *MLL2* [1]. Among those genes, *KDM6A* was inactivated in 18% of the pancreatic cancer patients. These findings further demonstrated that characterization of these epigenetic alterations will advance our understanding of the mechanisms contributing to pancreatic tumorigenesis, and lead to new discoveries of diagnostic and prognostic markers and therapeutic targets. We have performed a systemic search of the literature published in the past 15 years related to epigenetic regulations in pancreatic neoplasms using PubMed. This review provides a brief update on the current knowledge of alterations of epigenetic regulators in pancreatic neoplasms and their clinical implications (Table 1).

## 2. DNA Methylation

DNA methylation is one of the key mechanisms of epigenetic regulation. Genome-wide DNA methylation mapping enabled insights into the dynamics of DNA methylation. It is known that DNA methylation at gene promoter CpG islands blocks transcription initiation, whereas DNA methylation in gene body may facilitate transcription elongation and different splicing. DNA methylation is frequently found in repeat-rich areas of the genome and is essential for genomic and chromosomal stability [6]. DNA methylation is also critical for maintaining pluripotency, X-chromosome inactivation and genomic imprinting [6]. Aberrant DNA methylation is the best characterized epigenetic alteration in cancer [7]. Cancer cells tend to lose global and gene-specific DNA methylation. In addition, DNA methylation of tumor suppressor gene promoters is thought to be a major epigenetic mechanism in tumorigenesis. Gene promoters of *APC*, *BRCA1*, and *p16INK4a* are among the most frequently methylated genes in human pancreatic neoplasms [7]. Aberrant gene methylation involves the genes in the TGF-β, WNT, integrin, Slit Guidance Ligand (SLIT)-Roundabout Guidance Receptor (ROBO) signaling, cell adhesion, and stellate cell activation pathways.

DNA methylation is carried out by the only known enzyme DNA methyltransferases (DNMTs). DNMTs transfer a methyl group from *S*-adenosylmethionine (SAM) to the fifth carbon of cytosine residues in DNA [8]. Besides the CG sites, DNMTs also methylate CA dinucleotides [9]. There are three active mammalian DNMTs (DNMT1, DNMT3A and DNMT3B), and one regulatory protein, DNMT3L. DNMT1 is a maintenance DNA methyltransferase that preserves methylation patterns during cell division. It preferentially methylates CG dinucleotides through its interaction with UHRF1 [10]. DNMT3 enzymes induce de novo methylation during embryonic development. The intrinsic sequence preferences of DNMT3 enzymes are important for global de novo methylation, whereas crosstalk with other factors appears to be essential for locus-specific DNA methylation [11]. DNMT3L has no methyltransferase activity, but it stabilizes the active-site loop of DNMT3A to enhance de novo methylation and increases the binding of SAM [12]. In addition, genome-wide analyses have revealed an inverse correlation between DNA methylation and histone H3K4 methylation and a strong correlation between DNA methylation and H3K36me3, which suggests that DNMTs can recognize histone modifications and be recruited to specific nucleosomes [13]. DNMTs also cross talk with H3K9me3 and the repressive mark H3K27me3 [14].

A class of small non-coding RNAs (ncRNAs) called the Piwi-interacting RNAs (piRNAs) was found to guide de novo DNA methylation to specific sequences in mammals [15]. piRNAs are short 26–31 nucleotides RNAs that are predominantly expressed in mammalian germlines. They are characterized by their association with proteins in the Piwi subfamily of Argonaute proteins. Studies have shown that DNMT3A and DNMT3B are able to interact with small ncRNAs and form RNA protein complexes [16,17]. Another type of ncRNA, promoter-associated RNA (pRNA), was shown to bind to the promoter of rRNA genes and the DNA: RNA triplex was recognized by DNMT3B in mouse embryonic fibroblasts [18]. Furthermore, a few miRNAs (miR-29 family and miR-148) were implicated in targeting *DNMTs* for degradation or decreased expression through other transcription factors [8]. These studies suggest that ncRNAs have a critical role in guiding DNMTs to specific genomic loci.

Recent studies have found that aberrant DNA methylation contributes to critical signaling pathways involved in pancreatic tumorigenesis. For example, TGF-β promotes epithelial to mesenchymal transition (EMT) of pancreatic cancer cells partially by inducing hypermethylation of CpG site in *VAV1* gene body and VAV1 expression [19]. LKB-1/STK11 loss sensitizes pancreatic tumor cells to DNA methylation and inhibition of serine biosynthesis, and thus connecting cancer metabolism to DNA methylation and tumorigenesis [20].

Multiple studies have investigated altered DNA methylation pattern in pancreatic neoplasms. Guo et al. examined 48 pancreatic exocrine and endocrine neoplasms, including acinar cell carcinomas, PDAs, and neuroendocrine tumors, for DNA methylation changes of specific gene promoter regions and found that the six most frequently methylated genes are *APC* 50%, *BRCA1* 46%, *p16INK4a* 35%, *p15INK4b* 35%, *RARβ* 35%, and *p73* 33% [21]. Overall, 94% of the tumors had methylation of at least one gene. Furthermore, PDAs had different patterns of gene methylation from neuroendocrine tumors. Nones et al. assessed DNA methylation in 167 untreated resected PDAs and compared them to 29 adjacent nontransformed pancreatic tissue and found a list of 3522 genes differentially methylated [22]. Atypical methylation occurred in genes involved in important molecular mechanisms, including TGF-β, WNT, integrin, cell adhesion, stellate cell activation, and axon guidance signaling pathways. The aberrant methylation of some of the genes, such as *BNC1* and *ADAMTS1*, was shown to be detected in the serum of patients with PDA, which could be served as potential early diagnostic biomarkers for PDAs [23]. Cell-free DNA promoter hypermethylation in patient plasma was also shown to be significantly different between pancreatic cancer patients and control group [24]. Another study has examined DNA methylation differences in leukocyte DNA between PDA patients and controls [25]. The authors found significant methylation differences in 110 CpG sites and using a combination of 5 CpG sites, they were able to discriminate PDA patients from controls. These studies suggest that DNA methylation is altered in PDAs, which could serve as potential diagnostic or prognostic biomarkers for PDAs.

Increased expression of DNMT1, DNMT3A and DNMT3B was reported in pancreatic cancer, suggesting their role in pancreatic cancer development. Wang et al. examined the expression level of DNMT1 by immunohistochemistry staining in PDAs, benign pancreatic tissues, and PDA precursor lesions (pancreatic intraepithelial neoplasia (PanINs) and intraductal papillary mucinous neoplasm (IPMN)), and found that DNMT1 expression levels increased from benign tissue to precursor lesions to PDA [26]. The expression of DNMT1 also significantly correlated with perineural invasion, tumor differentiation, and staging, suggesting that DNMT1 could be prognostic markers for PDAs. Another study evaluated DNMTs in 88 PDAs and 10 normal pancreatic tissues, and found that DNMT1, DNMT3A and DNMT3B proteins were expressed in 46.6%, 23.9%, and 77.3% of PDA tissues, respectively, but not in normal pancreatic tissues [27]. DNMT1 expression is also associated with poor prognosis in PDA patients. DNMT1 was found to interact with menin, which is frequently mutated in pancreatic neuroendocrine tumors, and reversibly regulates pancreatic cancer cell growth [28]. Quantitative real-time RT-PCR was used to detect *DNMT* expression and showed that the mRNA expression levels of *DNMT1*, *DNMT3A*, and *DNMT3B* increased from normal ducts to PanINs and then to PDAs and correlated with staging [29]. *DNMT3A* and *DNMT3B* expression also correlated with tumor size [29]. These studies demonstrated that DNMTs are potential diagnostic and prognostic markers, and therapeutic targets for pancreatic cancer.

Two types of small molecule DNMT inhibitors have been developed, namely, nucleoside and non-nucleoside [30]. Nucleoside analogs based on epigenetic inhibitors 5-azacytidine and 5-aza-2′-dC are in Phase I-III clinical trials for many human diseases, and two DNMT inhibitors, azacytidine and decitabine, have shown efficacy and received FDA approval for the treatment of myelodysplastic syndrome but not solid tumors [31,32]. Nevertheless, some studies showed 5-aza-2′-dC decreased pancreatic cancer cell proliferation and induced cell cycle arrest in an in vitro model [33,34,35]. Recent pre-clinical studies also showed that DNMT inhibitors enhance the efficacy of PARP inhibitors in acute myeloid leukemia and breast cancer cells [36]. 5-azacytidine preferentially incorporates into RNA while 5-aza-2′-dC incorporates into DNA, which leads to higher potency with 5-aza-2′-dC and more off-target effects with 5-azacytidine [37,38].

## 3. Histone Modifications

Perhaps one of the most interesting epigenetic regulators in pancreatic cancer oncogenesis is histone modification, especially histone methylation. Most recent findings from the whole genomic sequencing data revealed that some of the most frequently mutated epigenetic genes in pancreatic cancer belong to this family. Post-translational modifications of histones include acetylation, methylation, phosphorylation, ADP ribosylation, deamination, ubiquitination, and sumoylation [39]. These histone modifications may alter chromatin structure and regulate important cellular processes such as transcription, replication, or DNA repair. Histone modifications are a dynamic process that is usually carried out by pairs of enzymes with reverse catalytic functions, such as histone acetyltransferases (HATs) and histone deacetylases (HDACs). The balance between these enzymes is essential for maintaining normal cellular function. Studies have found that alterations of many histone modification enzymes are associated with human diseases, including cancer [39]. Histone acetylation and methylation are the two most characterized mechanisms in pancreatic tumorigenesis.

### 3.1. Histone Methyltransferases and Demethylases

Histone methylation, which is regulated by histone methyltransferases (HMTs) and histone demethylases (HDMs), occurs on the lysine, arginine, and histidine residues at the amino acid side chains. Lysine methylation is the most characterized and is regulated by histone lysine methyltransferases (HKMTs). Depending on which lysine residue is methylated, the result could be either transcription activation or silencing. Additionally, each residue can be mono-, di-, or tri-methylated, which provides another layer of regulation. In contrast, lysine-specific demethylases (KDMs) remove the methyl group from histone.

**Figure 1:**
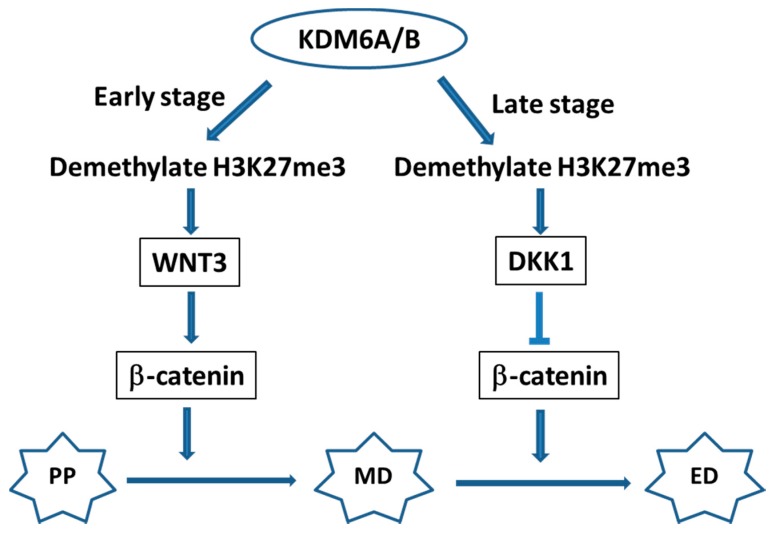
Proposed model of the role of KDM6A/B in definitive endoderm differentiation. At early stage of definitive endoderm differentiation from human embryonic stem cells, KDM6A/B demethylate H3K27me3 which leads to upregulation of WNT3 expression and activation of WNT signaling pathway, which further promotes mesendoderm differentiation. At late stage, KDM6A/B activates DKK1, a WNT antagonist, to suppress WNT signaling pathway and promote mesendoderm to endoderm differentiation. Arrow indicates activation and T-bar indicates inhibition. PP: pluripotent; MD: mesendoderm; ED: endoderm.

The two most frequently altered histone methylation regulatory genes in pancreatic cancer are *KDM6A* and *MLL2* [1]. KDM6A is an H3K27me3 demethylase. Studies show that KDM6A is crucial in endoderm differentiation, including pancreas, from human embryonic stem cells by regulating WNT signaling pathway and *HOX* gene expression (Figure 1) [40,41]. At early stage of definitive endoderm differentiation, KDM6A/B demethylate H3K27me3 which leads to upregulation of WNT3 expression and activation of WNT signaling pathway, which further promotes mesendoderm differentiation. At late stage, KDM6A/B activates DKK1, a WNT antagonist, to suppress WNT signaling pathway and promote mesendoderm to endoderm differentiation (Figure 1).

Enrichment of H3K27me3 mark on the promoters of several genes is associated with reduced expression of these genes in Brg1-depleted IPMN-PDA cells [42]. Whole genome sequencing and copy number analysis recently discovered *KDM6A* as a new candidate driver of PDA [1]. Most of the variations of *KDM6A* in PDAs are loss of copy number with a few cases of single nucleotide variation, in-frame deletion, or amplification, suggesting a potential tumor suppressive role in PDA. MLL2 is an H3K4 methyltransferase and was also shown to be altered in a small percentage of PDAs [1]. The genomic landscape of PDA cell lines also uncovered enrichment of positive epigenetic marks of H3K4me1 and H3K4me3. EZH2, a H3K27 methyltransferase and a component of polycomb group protein complex PRC2, has been shown to be overexpressed in pancreatic cancer cell lines and 68% of PDAs [43]. Nuclear localization and high expression of EZH2 was associated with poorly-differentiated PDAs and shorter survival in PDA patients, therefore it can be useful as a prognostic biomarker in PDA [44]. G9a is another HMT that was found to be overexpressed in PDAs and promote tumor invasion and metastasis [45]. Studies found that G9a controls cancer metabolism by epigenetically activating the serine-glycine synthesis pathway to support cancer cell proliferation [46].

Many HMT inhibitors have been developed and are in preclinical or clinical trials to treat hematologic malignancies such as acute myeloid leukemia [47]. Examples include DOT1L (H3K79 methyltransferase) inhibitors EPZ4777 and EPZ5676, PRMT1 (H4R3 methyltransferase) inhibitor AMI-408, EZH2 inhibitors DZNep and UNC1999, KDM1A (H3K4 demethylase) inhibitors GSK2879552, ORY-1001, and SP2509, and KDM4C (H3K9 demethylase) inhibitor SD70. EZH2 inhibitor DZNep has been shown to enhance the treatment effect of gemcitabine in PDA cell lines and PDA primary tumor cell cultures [48]. More potent and selective small molecule inhibitors of EZH2, such as GSK126, EPZ-6438, UNC-1999 and CPI-169, are being developed for potential candidate therapeutics for PDA [49]. The KDM family is also an optimal pharmacological target. GSK-J1/J4 was the first potent KDM6 inhibitor developed and has been shown to be effective in glioma xenografts [50,51]. However, later studies have found that this compound may not be very selective for KDM6A/B since it also inhibits KDM5B/C [52]. New and improved KDM1A inhibitors that belong to the *N*′-(1-phenylethylidene)-benzohydrazides have also been identified by structure-based computer-simulated screenings and were shown to inhibit cell proliferation of several cancer cell lines [53]. BRD4770 is a small molecule inhibitor of G9a that induces pancreatic cancer cell death by autophagy [54].

### 3.2. Histone Acetyltransferase and Deacetylase

Histone acetylation is the first discovered and also the most widely studied histone modification [55]. The acetylation of histone at lysine residue neutralizes its positive charge and weakens histone-DNA interaction, which generally leads to relaxed chromatin and transcriptional activation. HATs and HDACs are two families of enzymes that reversibly regulate this process. HATs transfer an acetyl group from acetyl-coenzyme A to positively charged ε-amino group of lysine residues, while HDACs remove the acetyl group. HDACs are classified into four classes based on homology to yeast HDACs. Several transcriptional coactivators including SRC-1, cAMP-response element binding protein (CBP)/p300 and general control of amino acid synthesis 5 (GCN5)/PCAF have HAT activity [39]. On the other hand, HDACs often suppress transcription and many transcriptional corepressors such as mSin3A and Mi-2/NuRD have HDAC activity. Dysregulation of HATs and HDACs is associated with many human diseases including cancer. As an example, global loss of acetylation at H4K16 is a common hallmark of human cancer [56].

Altered HATs and HDACs expression are associated with pancreatic neoplasms. One study showed a significantly decreased expression of p300, a HAT, secondary to up regulation of several miRNAs, in highly metastatic PDA cell lines [57]. Inactivating missense mutation of *p300* was also observed in a PDA cell line, providing evidence for the tumor suppressive role of *p300* in PDA [58]. Abnormal expression of HDACs has been more frequently reported in PDAs. HDAC2 and HDAC7 expressions are increased in PDAs, especially in poorly-differentiated cases [59,60]. Increased expression of HDAC7 distinguishes PDA from other benign pancreatic neoplasms and thus can be a potential biomarker for PDA. HDAC1 was also found at increased levels in 56% of the PDAs and its precursor lesions, and was required for pancreatic epithelial proliferation [61,62]. HDAC1 was shown to play a key role in the uncontrolled proliferation during pancreatic tumorigenesis. In zebrafish, a loss-of-function mutation in *HDAC1* resulted in impaired cell cycle progression in pancreatic epithelia. In another study, class I HDACs (HDAC 1–3) were found to be overexpressed in a subset of PDAs and there was a correlation with increased nuclear localization of RelA/p65 [63]. In addition, class I HDACs mediate the cross talk between p53 and NF-κB in cancer cells [64].

Inhibitors of both HATs and HDACs have been explored to target hematologic and solid tumors, including pancreatic cancer [65,66]. HAT inhibitors range from less specific natural substances to covalently modified isothiazolones [67]. Most of the HAT inhibitors target CBP/p300 and almost all are in preclinical phase, except curcumin which is in clinical trial [68]. HDAC inhibitors are probably the best characterized epigenetic drugs used in cancer therapies. HDAC inhibitors have been shown to reactivate tumor suppressor gene expression and lead to decreased cell proliferation and apoptosis [69]. However, targeting HDACs are more complicated since HDACs often form various multiprotein complexes with other proteins, including other HDAC family members, and target either tumor suppressors or oncogenes [70]. The current HDAC inhibitors inhibit their enzymatic activity regardless of the complex. Therefore, the outcome of these drugs is not predictable and probably needs further careful studies and extensive trials. Nevertheless, so far the HDAC inhibitors seem to be well tolerated and some have shown promising antitumor activity and entered preclinical or clinical trials, including abexinostat, pracinostat, resminostat, givinostat, panobinostat, and CUDC-101 [65].

### 3.3. Histone Ubiquitination

Histone ubiquitination has also been implicated in pancreatic homeostasis and pancreatic cancer tumorigenesis [71,72,73]. Histone ubiquitination mainly occurs on H2A and H2B and leads to mostly their mono-ubiquitination which does not lead to protein degradation but alters nucleosome dynamics and cross-talk between other histone modifications [74,75]. H2A ubiquitination is induced by DNA damage and subsequently leads to recruitment of downstream DNA damage repair proteins. H2B ubiquitination and deubiquitination are essential for double strand break repair and transcription-coupled repair, respectively. The major ubiquitination site on H2A was lysine residue 119 (K119) [76,77]. Protein ubiquitination requires the sequential activities of ubiquitin activating enzyme (E1), ubiquitin-conjugating enzyme (E2), and ubiquitin ligase (E3) [78]. Human Polycomb repressive complex 1 (PRC1)-like is responsible for H2A ubiquitination at K119 [79]. Among the three subunits of PRC1 (Ring1, Ring2, and Bmi-1), only Ring2 (Ring1B) is able to ubiquitinate H2A in vitro, suggesting that Ring2 is the E3 ligase for H2AK119. However, the presence of Ring1 and Bim1 greatly stimulates the ligase activity of Ring2 for H2A. Studies have found that over 50% of PDA tissues have increased expression of H2AK119 monoubiquitination (H2AK119Ub1) and Ring2 [73]. In addition, H2AK119Ub1 is an independent predictor of clinical prognosis. High expression of H2AK119Ub1 was found to be significantly associated with larger tumor size, poor differentiation, and lymph node metastasis [73]. Silencing of *Ring2* in PDA cells depleted H2AK119Ub1 and inhibited tumor cell growth. Other studies showed that Bim1 is required for the initiation of murine pancreatic neoplasia and is independent of the *Ink4a/Arf* locus [71]. In addition, Bim1 and Ring2 promote gene silencing through H2AK119Ub1 in acinar-to-ductal metaplasia and PDA cells [72]. These observations demonstrated the importance of histone ubiquitination in pancreatic cancer development.

## 4. Chromatin Remodeling

The ATP-dependent chromatin remodeling complexes are another group of important epigenetic alterations in pancreatic cancer with the SWItch/sucrose non-fermentable (SWI/SNF) complexes being the most studied and frequently altered. ARID1A, ARID1B, PBRM1, SMARCA2, and SMARCA4 are all components of the SWI/SNF complexes, and were shown by genomic sequencing studies to be mutated in pancreatic cancer [80]. The SWI/SNF complexes remodel chromatin through mobilization of nucleosomes both by sliding and by ejection or insertion of histone octomers and thus regulate gene transcription [81]. The SWI/SNF complexes target promoters and enhancers by mechanisms that are not fully understood. The two major families of SWI/SNF complexes in human are BAF and PBAF complexes that composed of 12–15 subunits with a set of core subunits [82].

About 20% of human cancers contain mutations in the subunits of SWI/SNF complex [82]. Furthermore, it is considered as a central tumor suppressive complex in pancreatic cancer [83]. Although mutations of the individual subunit do not occur at a high frequency, together they are altered in at least a third of the PDAs. Putative DNA binding subunits *ARID1A*, *ARID1B*, and *PBRM1*, enzymatic subunits *SMARCA2* and *SMARCA4*, and *ARID2* have been shown to be mutated in pancreatic cancer [1,82,83]. Promoter polymorphisms in the *SMARCA2* gene are strongly associated with pancreatic cancer patient survival [84]. BRM expression was shown to be related to tumor size, lymphatic invasion, and tumor stage [85]. Furthermore, high BRM and low BAF180 expression were also independent predictors for worse survival in the subgroup with adjuvant gemcitabine. Case control studies have also found significant association between some common variants in *BRD7* and *SMARCA4* and increased risk of PDA in the Chinese population [86]. Re-expression of SMARCA4 in SMARCA4-deficient pancreatic cancer cell lines reduced cell growth [83]. Loss of Brg1 cooperates with Kras to form cystic neoplasms in mouse models resembling human IPMN and progress to PDA [42,87]. Brg1 and Brm distinctly regulate Pdx1 activity in islet β cells [88]. Another SWI/SNF subunit, ARID1B, has significantly decreased expression in PDAs, especially in advanced-stage tumors, and ectopic expression of ARID1B led to attenuated colony formation in PDA cells [89]. These findings suggested that SWI/SNF subunits may be tumor suppressors. However, recent evidence suggested that some of the SWI/SNF subunits also have oncogenic activities [90]. Many mutations in SWI/SNF subunits lead to aberrant residual complexes formation, gain of oncogenic function, and promotion of tumor growth. For example, Brg1 inhibits the dedifferentiation prior to malignant transformation in mature pancreatic ductal cells but promotes tumor progression in fully-developed PDA [90]. Subunits of the SWI/SNF complex can also be overexpressed in several types of cancer. Additionally, knockdown of BRG1 inhibited PDA cell growth, supporting an oncogenic role of SWI/SNF subunits. Therefore, the role of the SWI/SNF complex is very context-dependent and its newly discovered oncogenic role in pancreatic cancer has gained attention as a potential target for cancer therapy.

The nature of the SWI/SNF complex demands selective targeting of the oncogenic activities of the complex. Currently targeting protein-protein interactions seems to be the most promising strategy since it is more selective. Some of the most promising strategies include targeting the inter-subunit interactions and interactions with other oncogenic transcription factors such as NF-κB or GLI1 [82]. A few selective bromodomain inhibitors are now in clinical trials for treatment of several cancers [91]. SWI/SNF subunits BRG1, BRM, BAF180, BRD7, and BRD9 contain bromodomains. Inhibitors selectively targeting BRG1/BRM, such as PFI-3, are being developed. Targeting the ATPase domain is another potential therapeutic approach since an ATP-binding pocket-deficient mutant cannot rescue the phenotype when BRM is knocked down. However, this is a challenging strategy since little structural data is available and it is rather nonspecific.

## 5. Non-Coding RNA

Non-coding RNAs are RNAs that are not translated into proteins. They include different classes of small RNAs and long non-coding RNAs (lncRNAs) whose expression is tissue and stage specific. In addition to translational repression and RNA degradation, studies have found that small RNAs modify chromatin structure and regulate gene expression by mediating histone or DNA methylation [92,93,94]. lncRNAs were recently described to regulate chromatin modifications and structure by recruiting chromatin-modifying complexes independent of small RNAs [95,96]. Here we review the implications of non-coding RNAs in PDA development.

### 5.1. Small RNAs

The RNA interference (RNAi) pathway plays an important role in early pancreas development including maintaining multipotent pancreatic progenitor cells (MPCs) and acinar cell differentiation since inactivation of the small RNA processing enzyme Dicer1 in MPCs and acinar cells led to pancreas agenesis and failure to establish acinar cell differentiation [97]. Several microRNAs (miRNAs) including Let-7b, miR-495, and miR-18a are also essential in regulating acinar cell differentiation and homeostasis by modulating the expression of transcription factors [98,99]. Furthermore, miRNAs such as miR-375, miR-7, and miR-26a are crucial for pancreatic endocrine cell differentiation by regulating transcription factor expression and DNA methylation [100,101,102].

Altered expression of miRNAs is common in PDA and its precursor lesions including IPMNs, suggesting their role in tumorigenesis. Examples include miR-21 and miR-155 which are upregulated in IPMNs and correlated with disease progression [103]. Loss of miR-101 upregulates EZH2, an H3K27 methyltransferase, and contributes to the carcinogenesis of IPMN [104]. In patients with PDA, 64 miRNAs were found to be deregulated according to a literature review [105]. Among those, increased expression of miR-21, miR-155, miR-196a-2, miR-203, miR-210, and miR-222 are associated with poor prognosis. A newer summary of miRNA alterations in PDA was provided in another recent review [66]. miR-34a is probably one of the most studied miRNAs that are decreased in PDA and data supports that it is an important tumor suppressor in PDA [106]. There are various mechanisms used to regulate miRNA expression including epigenetic mechanisms such as promoter methylation and transcription factor alterations [107]. Other studies suggested that miRNAs such as miR-125a also modulates chemo-resistance to gemcitabine in PDA through targeting A20 [108]. However, there is a lack of reproducibility in many of the deregulated miRNAs due to the differences in measurement, protocol, sample size, and variabilities among samples with a few exceptions.

miRNAs have been used as diagnostic and prognostic biomarkers for the early detection and prediction of outcome of PDA. miR-155 has been used in diagnosing IPMNs in pancreatic juice samples which showed higher miR-155 in 60% of IPMN cases and none of the control cases [109]. Similarly, miR-21 was also tested in pancreatic cyst fluid and was found to be a potential biomarker to differentiate benign, premalignant, and malignant pancreatic cystic lesions [110]. Circulating miR-25 was found to be a potential biomarker for early detection of PDA in patients’ serum with an impressive area under the ROC curve of 0.915 [111]. Plasma miR-22-3p, miR-642b-3p, and miR-885-5p were also shown to have high sensitivity in the diagnosis of early stage PDA together with CA19-9 [112]. A panel of four miRNAs (miR-21, -155, -196a, and -210) were tested in blood samples of PDA patients and reached a sensitivity and specificity of 64% and 89% to distinguish PDA from controls [113]. Other miRNAs such as miR-18a was also found to be significantly higher in PDA patient plasma samples than healthy people [114]. miRNAs levels are also associated with clinical outcomes of PDA. Plasma miR-21 level has been associated with poor clinical outcome in locally advanced PDA patients treated with chemotherapy followed by chemo-radiotherapy in a randomized phase II trial [115]. Plasma miR-221 level correlates with distant metastasis and unresectable status [116]. Serum miR-196a can distinguish resectable from unresectable PDA and predict overall survival [117]. Therefore, miRNAs are potential diagnostic and prognostic biomarkers of PDA and its precursor lesions.

There is no miRNA-based therapy clinical trial approved for PDA [118]. However, promising preclinical animal study data point to a strong therapeutic potential of miRNA-based gene therapy for PDA. A couple of miRNA-based therapy has entered clinical trials for the treatment of hepatitis C, liver cancer or other solid tumors (such as melanoma and renal cell carcinoma) and hematologic malignancies [119,120]. The main obstacle for miRNA-based gene therapy includes limited delivery, in vivo instability, lack of cell specificity, and nonspecific gene targeting. Miravirsen, a miRNA inhibitor targeting miR-122, is the first miRNA-based gene therapy product that achieved a clinical trial for the treatment of hepatitis C. miR-34a mimics (MRX34) is the first miRNA replacement therapy developed by miRNA Therapeutics to reach phase I clinical trial for the treatment of cancer. The miR-34a mimics were incorporated into a lipid-based nanoparticle named SMARTICLES [119]. Preclinical studies have shown efficient delivery of the lipid nanoparticles to the liver which led to tumor regression and prolonged animal survival. Therefore, miRNAs are also promising therapeutic agents for PDA.

### 5.2. LncRNA

In addition to small RNAs, lncRNAs may also play an important role in pancreatic endocrine cell maturation during development since there are more than 1000 lncRNAs found conserved in mouse and human islets and 55% of them are islet specific and developmentally regulated [121,122]. LncRNAs control β-cell function by regulating imprinted loci and gene expression [123]. LncRNA has also emerged as a major mechanism for PDA tumorigenesis by regulating important cellular behaviors such as cell proliferation, invasion, metastasis, and chemo-resistance [124]. A recent genome-wide association study has found a lncRNA, LINC00673, as a potential tumor suppressor whose germline variation is associated with increased PDA risk [125]. LncRNA H19 is another potential oncogenic lncRNA that was recently found to be overexpressed in PDA and associated with tumor grade and metastasis [126,127]. LncRNAs are also potential diagnostic and prognostic biomarkers in PDA. Linc-pint, a p53 induced lncRNA, is less abundant in the plamsa and tissue samples of PDA patients than those of healthy, adjacent tissues, ampullary carcinomas, and cholangiocarcinomas, suggesting the potential use of Linc-pint as a diagnostic marker for PDA [128]. Furthermore, low Linc-pint plasma levels are associated with tumor recurrence, and decreased tissue Linc-pint correlates with poor prognosis, supporting that Linc-pint levels may be useful for predicting patient outcome. Another lncRNA, uc.345 is upregulated in PDA tissues and correlates with depth of invasion, higher stage, and decreased overall survival, suggesting that it may be another potential useful prognostic marker for PDA [129]. Another study investigated 5 lncRNAs that associated with PDA (H19, HOTAIR, HOTTIP, MALAT1, and PVT1) for potential biomarkers in PDA and found that HOTAIR, HOTTIP, and PVT1 were significantly increased in PDA [130]. Furthermore, the salivary HOTAIR and PVT1 were also significantly higher in patients with PDA, suggesting their potential usage as non-invasive biomarkers for detecting PDA. Recently, a phase 1/2a clinical trial of intratumoral administration of BC-819, a DNA plasmid expressing diphtheria-toxin gene under the control of lncRNA H19 regulatory sequences, was carried out in patients with unresectable PDA and shown additional therapeutic benefit [131]. Nevertheless, therapeutic application of lncRNA in PDA is still in its infancy.

## 6. Conclusions

**Figure 2:**
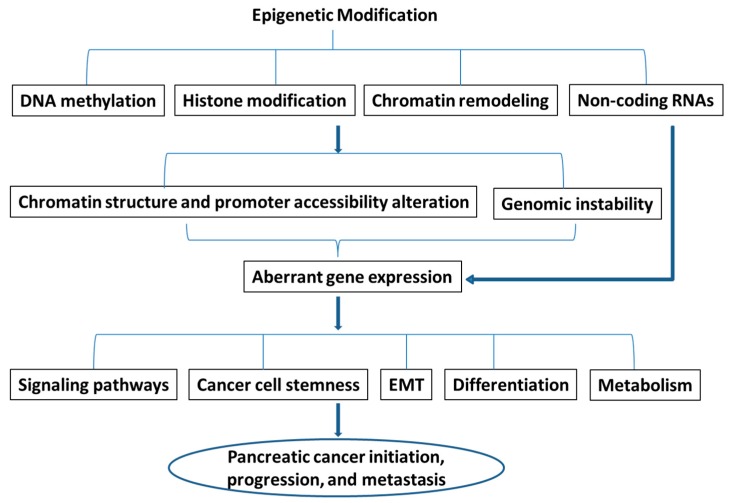
A diagram to summarize the role of epigenetic modifications in pancreatic cancer development and progression. Arrow indicates leading to or promoting. EMT: epithelial–mesenchymal transition.

In summary, it has become clear that distinct epigenetic events are sufficient to drive tumor formation, progression, and metastasis (Figure 2). Recently, numerous mutations have been found in chromatin modifier genes. Due to the broad coverage of these epigenetic regulators across the genome, their genetic alterations have major impact on vital cellular processes such as differentiation and proliferation. These mutations have been shown to have profound effect on tumor epigenetic regulation, leading to oncogenic transcriptional programs such as EMT, increased cancer cell stemness, and altered cellular metabolism, differentiation, and other important signaling pathways (Figure 2). Most of the mutations involving these epigenetic regulators are truncating mutations, either frameshift or nonsense mutations, although some are missense mutations [132]. It is most likely that these mutations are inactivating mutations. Some of the mutations were found in many types of human malignancies. Although most of the mutations occur in a relatively low frequency, it is believed that at least some of these mutations are driver mutations in cancer development. However, exactly how these mutations affect epigenetic reprogramming in different cell types and in different stages of tumor development is still not clear. New therapies are under development targeting epigenetic alterations (i.e., IDH1). However, relatively little is known about the implication of these epigenetic mutations in pancreatic cancer. There is no doubt that much needs to be done to better understand the mechanisms of epigenetic regulations in pancreatic cancer before translating these new discoveries into clinical practice.

## Figures and Tables

**Table 1:** Overview of epigenetic alterations in pancreatic ductal adenocarcinoma (PDA)—see text for details and references.

Epigenetic Mechanism	Protein/RNA	Alterations in PDA
DNA methylation	DNMT1, DNMT3A, DNMT3B	Increased expression
Histone modification		
Histone methylation	KDM6A	Loss of copy number, single nucleotide variation, deletion, amplification
	MLL2	Loss of copy number, single nucleotide variation, deletion
	EZH2	Increased expression
	G9a	Increased expression
Histone acetylation	p300	Decreased expression, missense mutation
	HDAC1-3, HDAC7	Increased expression
Histone ubiquitilation	H2AK119Ub1, Ring2, Bim1	Increased expression
Chromatin remodeling	ARID1A, ARID1B, PBRM1, SMARCA2, SMARCA4, ARID2, BRD7	Mutations
	SMARCA2	Promoter polymorphyisms, increased expression
	BAF180	Decreased expression
Non coding RNA		
Small RNAs	miR-21, miR-155, miR-196a-2, miR-203, miR-210, miR-222, miR-25, miR-22-3p, miR-642b-3p, miR-885-5p, miR-18a	Increased expression
	miR-101, miR-34a	Decreased expression
lncRNAs	LINC00673	Germline mutation
	H19, uc.345, HOTAIR, HOTTIP, PVT1	Increased expression
	Linc-pint	Decreased expression

## Abbreviations

RNA	Ribonucleic acid
PDA	Pancreatic ductal adenocarcinoma
DNA	Deoxyribonucleic acid
DNMT	DNA methyltransferase
DNA	Deoxyribonucleic acid
SAM	*S*-adenosylmethionine
ncRNA	Non-coding RNA
piRNA	Piwi-interacting RNA
pRNA	Promoter-associated RNA
PanIN	Pancreatic intraepithelial neoplasia
IPMN	Intraductal papillary mucinous neoplasm
HAT	Histone acetyltransferase
HDAC	Histone deacetylase
HMT	Histone methyltransferase
HDM	Histone demethylase
HKMT	Histone lysine methyltransferase
KDM	Lysine-specific demethylase
CBP	cAMP-response element binding protein
PRC1	Polycomb repressive complex 1
SWI/SNF	SWItch/sucrose non-fermentable
lncRNA	Long non-coding RNA
RNAi	RNA interference
MPC	Multipotent pancreatic progenitor cell
miRNA	microRNA
